# Effects of caffeine intake prior to stress cardiac magnetic resonance perfusion imaging on regadenoson- versus adenosine-induced hyperemia as measured by T1 mapping

**DOI:** 10.1007/s10554-017-1157-4

**Published:** 2017-05-25

**Authors:** R. van Dijk, D. Kuijpers, T. A. M. Kaandorp, P. R. M. van Dijkman, R. Vliegenthart, P. van der Harst, M. Oudkerk

**Affiliations:** 1 Center for Medical Imaging, University Medical Center Groningen, University of Groningen, Hanzeplein 1 EB 45, Groningen, The Netherlands; 2 Department of Cardiology, University Medical Center Groningen, University of Groningen, Groningen, The Netherlands; 3 Department of Cardiovascular Imaging, HMC-Bronovo, The Hague, The Netherlands

**Keywords:** Coronary artery disease (CAD), Cardiac magnetic resonance (CMR), T1 mapping

## Abstract

The antagonistic effects of caffeine on adenosine receptors are a possible cause of false-negative stress perfusion imaging. The purpose of this study was to determine the effects of coffee intake <4 h prior to stress perfusion cardiac magnetic resonance imaging (CMR) in regadenoson- versus adenosine-induced hyperemia as measured with T1-mapping. 98 consecutive patients with suspected coronary artery disease referred for either adenosine or regadenoson perfusion CMR were included in this analysis. Twenty-four patients reported coffee consumption <4 h before CMR (15 patients with adenosine, and 9 patients with regadenoson); 74 patients reported no coffee intake (50 patients with adenosine, and 24 patients with regadenoson). T1 mapping was performed using a modified look-locker inversion recovery sequence. T1 reactivity was determined by subtracting T1_rest_ from T1_stress_. T1_rest_, T1_stress_, and T1 reactivity in patients referred for regadenoson perfusion CMR were not significantly different when comparing patients with <4 h coffee intake and patients who reported no coffee intake (976 ± 4 ms, 1019 ± 48 ms, and 4.4 ± 3.2% vs 971 ± 33 ms, 1023 ± 43 ms, and 5.4 ± 2.4%) (p = 0.70, 0.79, and 0.40), and similar to values in patients without coffee intake undergoing adenosine CMR. In patients with <4 h coffee intake, T1_stress_, and T1 reactivity were significantly lower for adenosine (898 ± 51 ms, and −7.8 ± 5.0%) compared to regadenoson perfusion CMR (p < 0.001). Coffee intake <4 h prior to regadenoson perfusion CMR has no effect on stress-induced hyperemia as measured with T1 mapping.

## Introduction

Stress perfusion cardiac magnetic resonance imaging (CMR) is an excellent technique to diagnose myocardial ischemia with high diagnostic accuracy [1, 2]. The most widely used stress agent in stress perfusion CMR is adenosine. This vasodilator is an unselective agonist for at least four of the adenosine receptor subtypes and results in coronary hyperemia due to the binding with cardiac A_2A_ receptors [3, 4]. Another vasodilator agent that can be used in stress CMR is regadenoson. Because this selective A_2A_ agonist does not bind to the other adenosine receptor subtypes, this stressor can be used in patients with chronic obstructive lung disease [5–7]. A particular concern when using vasodilator perfusion CMR in the evaluation of myocardial ischemia are false negative results [8]. They are reported to occur in around 5–15% of cases [2, 9, 10], and results from a substudy of the CE-MARC study suggest that over one-third of false negative studies may be related to drug interactions and subsequent insufficient stress [8]. Caffeine plays an important role in stress perfusion imaging because of his antagonistic properties to coronary hyperemia. As caffeine binds to the same receptors, intake of coffee or other caffeine containing products shortly before stress CMR may interfere with the hyperaemic and hemodynamic response during both adenosine and regadenoson stress CMR.

In CMR, native (non-contrast) T1 mapping can be used for the quantification of myocardial water content, as a derivative measure of myocardial blood volume (MBV) [11]. This property of native T1 mapping enables the quantitative assessment of changes in myocardial water content during stress perfusion CMR. The difference between stress and rest T1 values can differentiate between ischemic, infarcted, remote, and normal myocardium [12]. Recently, we showed that T1 mapping can also identify patients who are stressed inadequately during adenosine perfusion CMR. Coffee intake less than 4 h prior to the examination caused an inversion of T1 reactivity during adenosine perfusion CMR [13]. So far no studies analyzed the effect of caffeine intake on regadenoson perfusion CMR. We hypothesize that regadenoson perfusion CMR is less effected by the antagonistic effect of caffeine due to the highly selective A_2A_ agonistic properties of regadenoson.

The purpose of this study was to determine the effects of coffee intake less the 4 h prior to stress perfusion CMR in regadenoson- versus adenosine-induced hyperemia.

## Materials and methods

### Patient selection

**Fig. 1:**
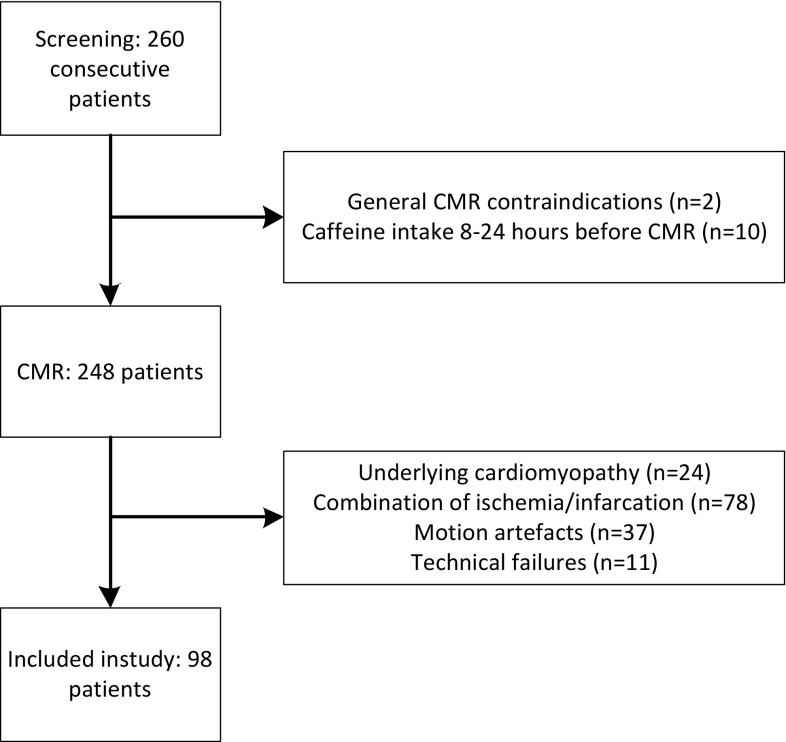
Flowchart of the inclusion and exclusion of patients. Motion artefacts were mainly due to incapability of patients with COPD to perform adequate breath holds

The institutional review board approved this study. All patients provided written informed consent before enrolment. Between August 2015 and March 2016, 260 consecutive patients with suspected coronary artery disease referred for stress CMR with either adenosine or, in case of contra-indications to adenosine (e.g., COPD), regadenoson were screened. All patients received prior, routine instructions, sent to their home, to avoid potential adenosine or regadenoson agonists for at least 24 h before CMR (e.g., coffee, tea, or chocolate and cacao). In addition, all anti-anginal medication (including beta-blockers) was discontinued 4 days prior to the examination. Patients on dypiridamole and unable to discontinue this drug were excluded. At arrival in the MRI facility patients were systematically interviewed on the recent intake of coffee and other caffeine containing substances. Visual assessment of the first pass perfusion images, motion artefacts, and Late Gadolinium enhancement images was performed. Patients were excluded from further analysis when ischemia, infarction, substantial motion artefacts, or technical failures were present A flow chart of the inclusion/exclusion is shown in Fig. 1. Results on rest-stress T1-mapping of the study population undergoing adenosine perfusion CMR was previously reported [13].

### CMR protocol

**Fig. 2:**
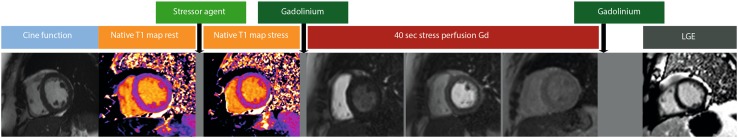
Overview of the cardiac CMR protocol. Cine function imaging is followed by native T1 mapping during rest and stress, 40 s stress perfusion imaging, and Late Gadolinium Enhancement images. Stressor agent was either adenosine (continuous infusion for 3 min before stress perfusion acquisition) or regadenoson (single bolus before stress perfusion acquisition)

**Fig. 3:**
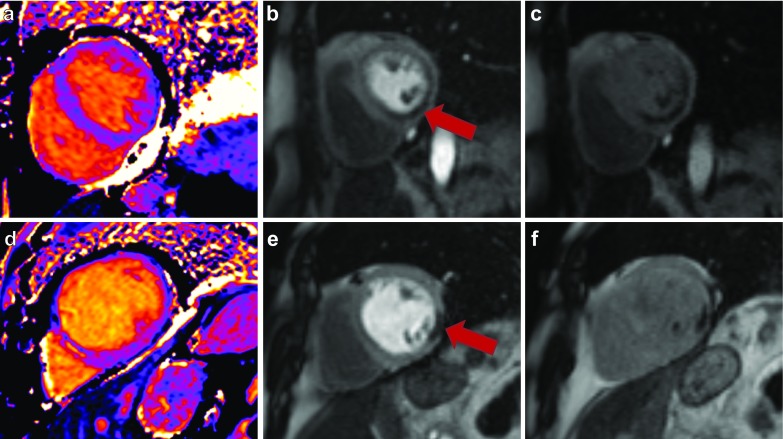
Myocardial ischemia assessment in two patients who consumed coffee several hours before Regadenoson perfusion CMR study. *Top row* 84 year-old women who had coffee < 4 h before the stress study (T1 reactivity of 4.6%). Native T1 mapping (**a**), peak perfusion (**b**), and late perfusion (**c**) images are shown. The perfusion image showed a transmural perfusion defect in the inferior wall in multiple segments, consistent with myocardial ischemia. This perfusion defect was caused by a significant stenosis in the right coronary artery, for which the patient received PCI treatment. *Bottom row* 75 year-old men who had coffee 2 h before the stress study (T1 reactivity of 4.2%). Native T1 mapping (**d**), peak perfusion (**e**), and late perfusion (**f**) images are shown. The perfusion study showed one segmental perfusion defect in the lateral wall. This patient was treated medically.

A 1.5T MRI system (Magnetom Avanto; Siemens Healthcare, Erlangen, Germany) was used in all patients. After the standard cine images, an investigational modified look-locker inversion recovery (MOLLI) based T1 mapping sequence (WIP780B) was performed at rest and in stress. A 5(3)3 sampling scheme of the heart was performed, including 8 images in 11 heartbeats. For the MOLLI acquisition an initial Inversion time of 110 ms was used with an 80 ms increment. A pixel-wise T1 map of the myocardium was acquired in a mid-ventricular short-axis view with inline motion correction. The images were generated using a single-shot steady-state free-precession readout. Typical parameters were: field of view, 300 × 256 mm²; slice thickness 8 mm; base resolution 192; in plane spatial resolution, 1.4 × 1.4 mm²; bandwidth, 1085 Hz/pixel; flip angle, 35° and parallel imaging acceleration factor, 2. Native T1 maps were acquired at rest, and 60 s after intravenous regadenoson injection (bolus of 400 µg, injected over 10 s). Next, stress-only perfusion imaging was performed as previously described [14]. In brief, a nonselective saturation recovery perfusion sequence was started during the first pass of 0.1 mmol/kg gadopentetate dimeglumine injected at a flow rate of 5 ml/s, directly after the stress T1 map. Equal position of the short-axis slices was used in T1 mapping and perfusion imaging. An overview of the CMR protocol is shown in Fig. 2. As a proof of concept we present two cases in which ischemia assessment was still possible despite of coffee intake prior to the regadenson perfusion CMR examination as shown in Fig. 3.

### Image analysis

T1 maps were independently analysed by two radiologists (DK, TK) with more than 10 years of experience in CMR. Both readers performed the analysis in a random order and were blinded to the patient data. The quantitative measurements of the T1 relaxation times were performed using commercially available mapping software (MASS analytical software, Medis, Leiden, The Netherlands). To avoid partial volume effects of the blood pool, all samples were in the core of the ROI, using conservative septal sampling [15]. For this study the perfusion and LGE series were not involved in the analysis (however, CMR examinations with signs of ischemia or infarction were at the beginning excluded).

### Statistical analysis

Continuous values are presented as mean values with ±standard deviation (SD). Categorical data are presented as percentages. Differences between two groups were assessed by using independent sample *t* test, and paired *t* test in case of paired data. One-way ANOVA was used to compare three or more groups. Statistical significance was defined as a p-value < 0.05. Statistical analyses were performed by using SPSS statistics 23 (IBM corporation, USA).

## Results

The total study population consisted of 98 consecutive patients, aged 65 ± 11 years, with 49% men. 24 patients reported consuming coffee <4 h (15 patients with adenosine, and 9 patients with regadenoson) before stress perfusion CMR and 74 patients who reported no coffee intake (50 patients with adenosine perfusion CMR, and 24 patients with regadenoson perfusion CMR). Patient characteristics and hemodynamics are shown in Table 1.

**Table 1:** Patient characteristics and hemodynamics

	Total (n = 98)	Adenosine control* (n = 50)	Regadenoson control (n = 24)	Adenosine caffeine <4 h* (n = 15)	Regadenoson caffeine <4 h (n = 9)
Age (years)	65 ± 11	67 ± 11	66 ± 11	67 ± 11	59 ± 11
Male	16 (49)	22 (44)	11 (46)	8 (53)	5 (56)
Systolic RR rest (mm Hg)	148 ± 20	146 ± 28	147 ± 19	144 ± 20	151 ± 21
Systolic RR stress (mm Hg)	135 ± 20	142 ± 23	133 ± 19	139 ± 15	139 ± 23
Diastolic RR rest (mm Hg)	88 ± 12	85 ± 19	89 ± 12	83 ± 9	85 ± 9
Diastolic RR stress (mm Hg)	81 ± 12	80 ± 10	82 ± 11	79 ± 7	79 ± 14
HR rest (bpm)	78 ± 18	76 ± 15	82 ± 18	70 ± 7	67 ± 12
HR stress (bpm)	100 ± 20	87 ± 14	107 ± 18	81 ± 10	84 ± 12
HRR^†^ (%)	21	16	32	16	26
Systolic RR response^††^ (%)	−5 ± 10	−2 ± 12	−9 ± 7	−3 ± 7	−8 ± 5
Diastolic RR response^‡^ (%)	−5 ± 10	−4 ± 11	−7 ± 9	−4 ± 8	−8 ± 10

^**†**^HRR—% change in HR from rest to stress

^††^Systolic RR response—% change in systolic RR

^‡^Diastolic RR response—% change in diastolic RR. Results are presented as mean ± standard deviation or n (%)

*Results as previously reported by Kuijpers et al. [14]

### T1 mapping in patients without self-reported coffee intake

T1_rest_, T1_stress_, and T1 reactivity values are shown in Table 2. In patients who reported no coffee intake T1_rest_, T1_stress_, and T1 reactivity were not significantly different when comparing the control adenosine group with the control regadenoson group (at p = 0.48, 0.62, and 0.08, respectively).

**Table 2:** Rest-Stress T1-mapping results

	Total (n = 98)	Adenosine Control* (n = 50)	Regadenoson control (n = 24)	Adenosine caffeine <4 h* (n = 15)	Regadenoson caffeine <4 h (n = 9)
T1 rest (ms)	975 ± 38	977 ± 41	971 ± 33	975 ± 42	976 ± 34
T1 stress (ms)	1001 ± 61	1018 ± 40	1023 ± 43	898 ± 51	1019 ± 48
T1-reactivity (%)	2.7 ± 5.5	4.3 ± 2.8	5.4 ± 2.4	−7.8 ± 5.0	4.4 ± 3.2

Results are presented as mean ± standard deviation

*Results as previously reported by Kuijpers et al. [14]

### T1 mapping in patients undergoing adenosine perfusion CMR

T1_rest_ in adenosine perfusion CMR was not significantly different when comparing patients with coffee intake <4 h prior to the examination with patients who reported no coffee intake (at p = 0.83). In contrast, T1_stress_, and T1 reactivity in patients with <4 h coffee intake were significantly lower as compared to patients without self-reported coffee intake (both at p < 0.001).

### T1 mapping in patients undergoing regadenoson perfusion CMR

T1_rest_, T1_stress_, and T1 reactivity in patients undergoing regadenoson perfusion CMR were similar for patients with coffee intake <4 h prior to the examination and patients who reported no coffee intake (at p = 0.70, 0.79, and 0.40, respectively).

### T1 reactivity mapping in patients with coffee intake <4 h prior to the examination

T1_rest_ in patients with self-reported coffee intake was similar for patients undergoing adenosine perfusion CMR and patients undergoing regadenoson perfusion CMR at p = 0.96. However, T1_stress_, and T1 reactivity were significantly lower in patients with <4 h coffee intake undergoing adenosine perfusion CMR when compared to patients with self-reported coffee intake undergoing regadenoson perfusion CMR both at p < 0.001.

### Heart rate and blood pressure

In the group of patients who reported no coffee intake (controls), resting heart rate (HR_rest_) was not significantly different when comparing adenosine perfusion CMR with regadenoson perfusion CMR (at p = 0.18). HR during stress (HR_stress_) was significantly lower in the control group of patients undergoing adenosine perfusion CMR as compared to the control group with regadenoson perfusion CMR (at p < 0.001). There was no significant difference in the diastolic blood pressure (RR) response when comparing the adenosine control group with the regadenoson control group (at p = 0.37). The systolic RR response and Heart rate response (HRR) were significantly lower in the adenosine control group as compared to the regadenoson control group (at p = 0.004, and p < 0.001).

There was no significant difference in HR_rest_ and HR_stress_ in patients who reported no coffee intake as compared to patients with coffee intake <4 h prior to the examination in the adenosine perfusion CMR group (at p = 0.16, p = 0.13). In the patients undergoing regadenoson perfusion CMR both HR_rest_ and HR_stress_ were significantly higher in patients without self-reported coffee intake when compared to the patients with coffee intake <4 h prior to the examination (at p = 0.03, and p = 0.002). The diastolic RR response, systolic RR response, and HRR in control patients undergoing adenosine perfusion CMR were not significantly different when compared to the diastolic and systolic RR response in patients with <4 h coffee intake at p = 0.96, 0.96, and 0.95. The diastolic RR response, systolic RR response, and HRR in patients without self-reported coffee intake undergoing regadenoson perfusion CMR were also not significantly different when compared to the diastolic and systolic RR response in patients with <4 h coffee intake at p = 0.81, 0.69, and 0.299, respectively.

## Discussion

Our study is the first to perform a direct comparison comparison of the effects of coffee intake prior to either adenosine or regadenoson stress perfusion CMR. To assess changes in myocardial blood volume, we determined native T1 values and we report that T1_stress_ and the T1 reactivity are not significantly affected by consumption of coffee in the hours prior to the stress perfusion CMR when regadenoson is used as stressor, in contrast to adenosine where coffee intake causes a strong negative effect. In the patients without coffee intake both stress agents induced a comparable increase in MBV as measured with T1 mapping and the ΔT1 values in these groups showed no significant difference with ΔT1 of the regadenoson caffeine <4 h group.

Previous research on the effects of caffeine ingestion on the response to vasodilator agents in stress perfusion imaging is limited and the available evidence shows contradictory results.

A study by Gaemperli et al. showed that, in positron emission tomography (PET) imaging, ingestion of a 200 mg caffeine capsule 2 h prior to regadenoson stress perfusion imaging had no significant effect on the pharmacological response as measured by the increase in myocardial blood flow during stress [16]. However, in a myocardial perfusion scintigraphy (MPS) study, there was a significant decrease in the number of segments with reversible defect in case of caffeine intake as compared to the placebo group in patients undergoing regadenoson MPS [17].

A possible explanation for our findings is the selective and potent A_2A_ nature of regadenoson as compared to adenosine. Regadenoson already achieves maximal coronary hyperemia when only 25% of the A_2A_ receptors is occupied [16]. Adenosine is a less potent and unselective A_2A_ agonist and thus potentially more sensitive to the competitive antagonistic properties of caffeine.

Secondly, a relatively high dose of caffeine (equivalent to 2–4 cups of coffee) was used in the regadenoson MPS study 90 min prior to the examination [17]. In our study serum caffeine measurement was not performed and patients did not received a fixed dose of caffeine. Coffee intake in our study was equivalent to 1–2 cups of coffee. As reported earlier, at lower concentrations caffeine clearance occurs through the principals of first-order elimination, however at higher dose zero-order elimination occurs [18, 19]. The differences in behaviour of caffeine at low and high dose is the most likely explanation for the findings of the discussed studies.

Animal studies investigating the effects of increasing concentrations of caffeine have shown a dose dependent relationship between caffeine and hemodynamic effects such as lowering of heart rate in the first hours after caffeine ingestion [20, 21]. In our study, patients undergoing adenosine perfusion CMR, showed no difference in the hemodynamic response when comparing patients without self-reported coffee intake to the patients with self-reported coffee intake. For the patients with regadenoson perfusion CMR both HR_rest_ and HR_stress_ were significantly lower in the group with coffee intake prior to the examination. These results are in conjunction with a study by Bitar et al., who reported a blunted rise in heart rate but no significant difference in HRR when comparing patients with and without caffeine ingestion [22]. The heart rate lowering effects of caffeine that we have observed in our study has been reported earlier [21, 23].

In our study the diastolic RR, systolic RR and HRR response in the patients undergoing regadenoson perfusion CMR were not significantly different when comparing patients with and without self-reported coffee intake. The differences in the hemodynamic response to either adenosine or regadenoson in combination with caffeine intake is possibly explained by interaction of the substances leading to a combination of altered sympathetic tone, variable chemoreceptor response, and vasodilatory effects [20].

### Limitations

This study was performed in a small study population with only self-reported coffee intake, meant as a proof of concept. Serum caffeine measurements were not performed and patients did not received a fixed dose of caffeine. We cannot exclude the possibility that a number of patients in our study has consumed other caffeine containing substances in the period before the examination. If so, this would have led to bias towards zero in the adenosine group, meaning that the measured effect that we found in our study is possibly an underestimation of the real effect of caffeine intake on hyperemia in adenosine perfusion CMR. Another limitation is the selection bias because only patients with contra-indications to adenosine, in this study COPD, received regadenoson as stressor. Due to the inability of these patients to perform an adequate breath hold, the MOLLI sequence used in this study showed many studies with motion artefacts, which could have been overcome by using shorter independent heart rate T1 sequences.

### Implications

Our study is the first to report on the effects of coffee intake prior to regadenoson perfusion CMR. We show that, in contrast to the negative effects during adenosine perfusion CMR, coffee intake has no effect on the hyperaemic response to regadenoson, as indicated by the T1 reactivity mapping.

This finding may be important in clinical practice, because regadenoson can be used directly as an adequate alternative stressor in patients referred for adenosine stress CMR who inadvertently ingested coffee before the stress study, reducing the number of cancelled and rescheduled stress studies.

Furthermore T1 reactivity, as an imaging biomarker, can be used as a bench-mark for the assessment of stress adequacy during vasodilator perfusion CMR and should be reported in the results.

## Conclusions

Coffee intake <4 h prior to regadenoson perfusion CMR appears to have no effect on stress induced hyperemia as measured with T1 mapping.

## Abbreviations

CAD: Coronary artery disease
COPD: Chronic obstructive pulmonary disease
HR: Heart rate
HRR: Heart rate response
LGE: Late gadolinium enhancement
MOLLI: Modified look-locker inversion recovery
CMR: Cardiac magnetic resonance
PET: Positron emitting tomography
PSIR: Phase sensitive inversion recovery
RR: Blood pressure
SPECT: Single photon emitting tomography
